# Relative normalized luciferase activity for the recombinant vector constructs carrying the ancestral and variant alleles for *XRCC2*:rs3218550 and *PHB*:rs6917

**DOI:** 10.1186/s13104-018-3760-4

**Published:** 2018-09-04

**Authors:** Nirmala Dushyanthi Sirisena, Nilakshi Samaranayake, Vajira H. W. Dissanayake

**Affiliations:** 1 Human Genetics Unit, Faculty of Medicine, University of Colombo, No. 25 Kynsey Road, Colombo 8, Sri Lanka; 2 Department of Parasitology, Faculty of Medicine, University of Colombo, Colombo 8, Sri Lanka

**Keywords:** Alleles, Breast cancer, Luciferase activity, Reporter genes, Single nucleotide polymorphisms, Vectors

## Abstract

**Objective:**

The data presented herein represents the preliminary results of the functional assays of a recently conducted larger study in which two single nucleotide polymorphisms (SNPs) [*XRCC2*:rs3218550 and *PHB*:rs6917] were significantly associated with risk of breast cancer among Sri Lankan postmenopausal women. The rs3218550 T allele and rs6917 A allele were found to increase breast cancer risk by 1.5-fold and 1.4-fold, respectively. Both SNPs are located in the 3′untranslated region (3′UTR) of the respective genes. It was hypothesized that these non-coding SNPs may be exerting some transcriptional regulatory effects on gene expression. Their putative functional effects were further investigated by generating bioluminescent recombinant experimental reporter gene constructs carrying the ancestral and variant alleles of these 2 SNPs, transiently transfecting them in MCF-7 breast cancer cell lines and performing dual-luciferase reporter gene assays to measure the luminescent signals.

**Data description:**

The normalized relative luciferase activity for the recombinant vector constructs carrying the ancestral and variant alleles for *XRCC2*:rs3218550 and *PHB*:rs6917 are presented herein. This data might be of relevance to other researchers involved in delineating the functional mechanisms of SNPs located in the 3′UTR of the *XRCC2* and *PHB* breast cancer related genes.

## Objective

Herein we present the preliminary functional assay data of a recently published larger case–control study, involving 350 Sri Lankan postmenopausal women with histologically confirmed invasive breast cancer (cases), and 350 healthy postmenopausal women (controls), in which common genetic polymorphisms in the *XRCC2* and *PHB* genes were associated with increased risk of sporadic breast cancer [1]. Another paper describing the genetic variants associated with the clinicopathological profiles in this cohort was also recently published [2].

Two single nucleotide polymorphisms (SNPs) [rs3218550:NC_000007.14:g.152646870C>T, X-ray repair cross-complementing gene-2 (*XRCC2*)/7q36.1; and rs6917:NC_000017.11:g.49404181G>A, prohibitin-1 gene (*PHB*)/17q21.33] showed the strongest evidence for association with breast cancer risk. The rs3218550 T allele and rs6917 A allele were found to breast cancer risk by 1.5-fold and 1.4-fold, respectively.

Both are non-coding SNPs located in the 3′untranslated region (3′UTR). The exact biological mechanisms by which they regulate breast cancer risk is unclear [3, 4]. Polymorphisms in the 3′UTR have been reported to be associated with various phenotypic effects due to their regulatory actions on gene and protein expression [5].

We hypothesized that these two SNPs may exert transcriptional regulatory effects. Their putative functional effects were investigated by generating recombinant experimental reporter gene constructs carrying the ancestral and variant alleles of each SNP, transfecting them in MCF-7 breast cancer cell lines and performing the dual-luciferase reporter gene assay to measure luminescent signals. Bioluminescent reporter genes provide an efficient method for the indirect measurement of relative rates of transcription. We believe the data generated might be of relevance to other researchers involved in delineating the functional mechanisms of SNPs located in the 3′UTR of the *XRCC2* and *PHB* breast cancer related genes.

## Data description

The data files 1 and 2 shown in Table 1 represent the normalized relative luciferase activity for the recombinant experimental vector constructs carrying the ancestral and variant alleles for *XRCC2*:rs3218550 and *PHB*:rs6917 SNPs, respectively [6]. The results are expressed as relative luminescence units (RLU) and the ratio between firefly luciferase/renilla luciferase provide the normalized luciferase activity for each vector. The normalized luciferase activity for the two control vectors, pGL3P vector lacking the insert (experimental empty vector) and the pGL3C vector containing the SV40 promoter and enhancer, are also given for each experiment. The triplicate results indicated for each vector represent the data obtained from three independent transfections of MCF-7 breast cancer cell lines and dual-luciferase assays performed under similar experimental conditions.

**Table 1:** Overview of data files

Label	Name of data file	File types (file extension)	Data repository and identifier (DOI or accession number)
Data file 1	Relative luciferase activity for the recombinant vector constructs carrying C and T alleles for rs3218550	MS Excel file (.xlsx)	Figshare [10.6084/m9.figshare.6854189] [6]
Data file 2	Relative luciferase activity for the recombinant vector constructs carrying G and A alleles for rs6917	MS Excel file (.xlsx)	Figshare [10.6084/m9.figshare.6854189] [6]
Data file 3	Sequences of 3′UTR inserts, primer sequences, partial electropherograms of final experimental vectors and vector diagrams	MS Word file (.docx)	Figshare [10.6084/m9.figshare.6854189] [6]

The recombinant experimental reporter gene constructs carrying the 116 bp 3′UTR insert of *XRCC2:*rs3218550 and 148bp 3′UTR insert of *PHB:*rs6917 were generated using the Gateway recombination cloning technology with Clonase II (ThermoFisher Scientific, USA). Samples which were previously identified as homozygous for the ancestral and variant alleles of rs3218550 and rs6917 were used as the PCR templates for the production of the inserts. The Gateway pDONR™221 donor vector and the pGL3P3′GW destination vector were used. Data file 3 shows the sequences of the 3′UTR vector constructs, primer sequences and vector diagrams.

Transient transfection of the recombinant experimental reporter gene constructs into cultured MCF-7 breast cancer cells (ECACC catalogue no. 86012803) was carried out using FuGENE HD transfection reagent (Promega, USA). Cells were plated at a density of 2 × 10^5^ in 12-well culture plates in complete medium and cultured for 24 h to attain 50–70% confluency before transfection. Each transfection experiment was carried out with the recombinant experimental vectors carrying the two alleles of rs3218550 and rs6916. In parallel, the pGL3P and pGL3C vectors (Promega, USA) were also transfected in each experiment. All vectors were co-transfected with the internal control vector (pRL-SV40) for normalisation purpose.

The transfection mixture for each well consisted of 1ug of experimental vector DNA and 10 ng pRL-SV40 vector along with FuGENE HD:vector DNA at a ratio of 3:1 in 50 ul complete medium, based on the optimized FuGENE HD protocol database (Promega, USA). The cultured MCF-7 cells were allowed to express the transfected DNA for 24 h. Dual-luciferase reporter assay (Promega, USA) was performed following passive lysis of the transfected cells. All luminescent signals were measured using the Glomax 20/20 Luminometer with dual auto injector system (Promega, USA).

## Limitations

- The small size of the datasets obtained in this study prevented the data from being used as part of a full research paper.
- The variability observed among the replicates could probably be due to variations in the amounts of reporter genes which may have entered into the cultured cells during the three independent transient transfection assays. In addition, as most transcription factors are present at low concentrations, only a small proportion of reporters entering the cell may receive the full complement of proteins needed for the proper functioning of the regulatory region under study. Such limitations are intrinsic to reporter gene assays.
- Transient recombinant experimental reporter genes are not always an appropriate assay to assess the influence of small differences due to SNP variation on the transcriptional activity of a particular gene, partly due to the lack of an appropriate chromatin confirmation.
- The functional assays were designed to check the regulatory effects of the individual SNPs and not the effect of haplotypes.
- The experiments were not replicated with change in variables such as cell line being used and the type of promoters present in the vectors to fully explore the potential regulatory effects.

## Abbreviations

3′UTR: 3′untranslated region
*PHB*: prohibitin-1
SNP: single nucleotide polymorphisms
*XRCC2*: X-ray repair cross-complementing gene-2
